# Custodial Grandfamilies: Systemic Inequities, Cultural Resilience, and Trauma-Informed Interventions

**DOI:** 10.1093/geroni/igaf122.885

**Published:** 2025-12-31

**Authors:** Danielle Nadorff, Deborah Whitley

## Abstract

Custodial grandparents face intersecting systemic, cultural, and psychological challenges that require culturally responsive, equity-driven interventions. This symposium integrates four studies to advance research and practice. First, a mixed-methods study (Shillingsburg) identifies custodial grandparents’ preferences for trauma-informed parenting interventions (e.g., PC-Care), emphasizing relational skill-building and accessibility. Second, Littleton’s analysis of National Survey of Children’s Health data reveals rural-urban disparities in social determinants of health, with rural grandparents disproportionately facing barriers in neighborhood built environments. Third, a cross-cultural phenomenological study (Lee & Mendoza) compares Korean and U.S. grandparents through role theory, demonstrating how cultural norms shape caregiving experiences: Korean grandparents report role conformity and U.S. grandparents role conflict, yet both groups exhibit resilience during the pandemic. Finally, McLin’s survey of ethno-racially marginalized grandparents uncovers a counterintuitive link between resilience and prospective memory failures (objective lapses in remembering tasks), where resilience directly predicts more frequent failures—a relationship potentially explained by heightened cognitive load. However, physical activity mediates this pathway, reducing failures among grandparents with strong ethnic identity resolution (commitment to one’s identity). Collectively, these talks highlight: 1. The need for trauma-responsive interventions addressing systemic barriers (e.g., rural service gaps, legal advocacy); 2. Cultural humility in designing supports (e.g., role expectations in collectivist vs. individualist contexts); 3. Strengths-based approaches that leverage grandparents’ resilience and racial/ethnic identity while mitigating structural inequities. The symposium calls for policies and programs that bridge gaps between evidence-based practices and the lived realities of custodial grandfamilies, prioritizing accessibility, identity affirmation, and multi-level support systems. Grandparents as Caregivers Interest Group Sponsored Symposium

